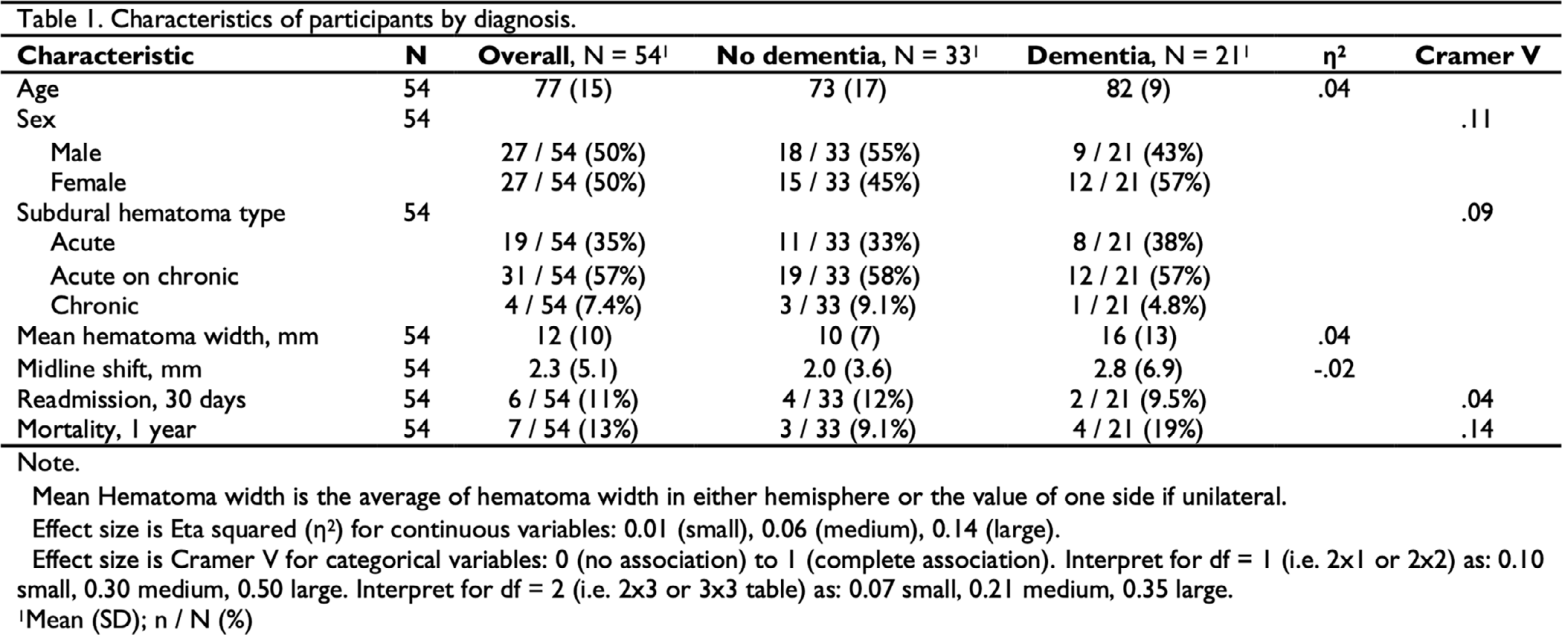# Effect of dementia on 1‐year mortality in subdural hematoma: a preliminary analysis

**DOI:** 10.1002/alz70861_108834

**Published:** 2025-12-23

**Authors:** M. Amin Banihashemi, Alireza Karandish, Dhrumhil Vaishnav, Muhammed Amir Essibayi, David J. Altschul

**Affiliations:** ^1^ Institute of Medical Sciences, The Temerty Faculty of Medicine, University of Toronto, Toronto, ON Canada; ^2^ Montefiore Medical Center, Albert Einstein College of Medicine, Bronx, NY USA

## Abstract

**Background:**

Aging and brain atrophy are risk factors for subdural hematoma (SDH). This preliminary analysis aims to quantify the effect of living with dementia on 1‐year mortality following hospital admission for SDH.

**Method:**

Medical record data between Dec 2021 and Jan 2023 were reviewed retrospectively with a sampling rate of 1:2 (with dementia: without dementia) for participants with SDH managed conservatively. Propensity score matching (PSM) was used to estimate the average marginal effect of living with dementia on 1‐year mortality, accounting for covariates (age, sex, chronicity of SDH, average hematoma width, midline shift). We used a 1:1 nearest neighbour PSM without replacement with propensity scores estimated using logistic regression of dementia status on covariates. Risk Ratio (RR) with 95% confidence intervals (CI) is reported from logistic regression after PSM to evaluate the association between dementia (as the sole predictor due to a low event rate <10) and 1‐year mortality, with statistical significance considered at *p* < 0.05.

**Result:**

A total of 54 adults with a mean age of 77±15 were included, 50% (27/60) being men, 39% (21/54) having dementia, and 7.4% (4/54) having chronic SDH. The 1‐year mortality rate was approximately twice in those living with dementia, 19% (4/21) vs. without 9.1% (3/33) (Cramer V = .14, df = 1, N = 54). After PSM, 12 non‐dementia cases were excluded, and all covariate differences were 0.3, indicating an acceptable balance for preliminary analysis. The effect of dementia on 1‐year mortality is as follows: RR = 1.33, 95% CI [0.28, 6.18], *p* = 0.713, N = 42.

**Conclusion:**

1‐year mortality appears 33% more likely in the dementia cohort, but the confidence interval is too wide to be conclusive. A sample size of 154 is needed for sufficient power (1‐β=0.80, α=0.05) and to include covariates with an absolute std. mean difference of > 0.1 after PSM.

**Additional information**: Funded by the Canadian Institutes of Health Research Micheal Smith Foreign Study Supplement (Funding reference number: FSS ‐ 191081)